# Potential activity of adiponectin‐expressing regulatory T cells against triple‐negative breast cancer cells through the cell‐in‐cell phenomenon

**DOI:** 10.1111/1759-7714.14940

**Published:** 2023-05-23

**Authors:** Wakana Chikaishi, Toshiya Higashi, Hirokatsu Hayashi, Yuki Hanamatsu, Yusuke Kito, Manabu Futamura, Nobuhisa Matsuhashi, Chiemi Saigo, Tamotsu Takeuchi

**Affiliations:** ^1^ Department of Gastroenterological Surgery and Pediatric Surgery Gifu University Graduate School of Medicine Gifu Japan; ^2^ Department of Pathology and Translational Research Gifu University Graduate School of Medicine Gifu Japan; ^3^ Department of Breast Surgery Gifu University Hospital Gifu Japan; ^4^ The United Graduate School of Drug Discovery and Medical Information Sciences Gifu University Gifu Japan; ^5^ Center for One Medicine Innovative Translational Research (COMIT) Gifu University Gifu Japan

**Keywords:** adiponectin, adoptive cell therapy, cell‐in‐cell, Treg, triple‐negative breast cancer

## Abstract

**Background:**

A population of regulatory T cells (Treg), which reside within thymic nurse cell complexes, express adiponectin and abrogate breast cancer development in transgenic mice. In this study, we examined whether adiponectin‐expressing Treg could impair triple‐negative breast cancer, which is defined by a lack of estrogen receptors, progesterone receptors, and human epidermal growth factor receptor‐2.

**Methods:**

CD4‐ and CD25‐positive cells were sorted from cultured T lymphocytes of a previously characterized experimental thymic tumor model composed of thymic nurse cells and abundant lymphoid stroma. These sorted cells were examined for FOXP3 and adiponectin immunoreactivity and subsequently exposed to triple‐negative breast cancer MDA‐MB‐157 and ‐231 cells.

**Results:**

Adiponectin‐expressing Treg were obtained by CD4‐ and CD25‐positive sorting and cell death was induced in triple‐negative breast cancer cells through the cell‐in‐cell phenomenon.

**Conclusions:**

Adiponectin‐expressing Treg may be candidates for adoptive cell therapy against triple‐negative breast cancer.

## Graphical abstract

Adiponectin‐expressing Treg induced cell death of triple‐negative breast cancer cells through the cell‐in‐cell phenomenon. Triple‐negative breast cancer cells were occulted with adiponectin‐expressing Treg. The cytoplasm of cancer cells was stained red, engulfed adiponectin‐expressing Treg stained green. Horizontal and vertical cell‐in‐cell images are shown in the upper left and lower left, respectively. Numerous adiponectin‐expressing Treg attached and integrated into cancer cells (upper right) subsequently destroyed cancer cells (lower right).

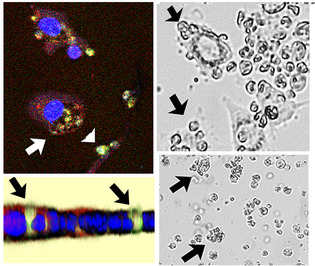

## INTRODUCTION

Triple‐negative breast cancer (TNBC) is a type of breast cancer characterized by the loss of estrogen receptor, progesterone receptor, and human epidermal growth factor receptor‐2 (HER2). Accordingly, they are not sensitive to endocrine therapy or HER2 treatment.^1^ Moreover, TNBC often demonstrates aggressive clinicopathological behavior; therefore, the standardization of treatment regimens for patients with TNBC is still in progress.^2^

Obesity also increases the risk of postmenopausal breast cancer.^3^ Recent advances have highlighted that adiponectin, a well‐characterized insulin‐sensitizing adipokine, and its receptors, especially T‐cadherin (also known as H‐cadherin or CDH13), may resist obesity‐related carcinogenesis.^4^ Paradoxically, the serum concentration of adiponectin is reduced in obesity.^5^ The impaired expression of adiponectin or adiponectin receptors leads to mammary gland carcinogenesis.^6^, ^7^, ^8^

Adiponectin induces autophagic cell death^9^ and can induce apoptosis via fatty acid metabolic reprogramming in breast cancer.^10^ However, T‐cadherin, a receptor for high‐molecular weights (HMW) adiponectin,^11^ which is known as a highly active form of adiponectin, is often lost in breast cancer cells due to promoter hypermethylation,^6^, ^12^, ^13^ worsening the prognosis of patients with TNBC.^14^ Therefore, an approach for the efficient introduction of adiponectin into breast cancer cells should be developed.

A population of Treg cells residing within thymic nurse cell complexes expresses adiponectin.^15^, ^16^, ^17^ Adiponectin‐expressing Treg significantly reduced mammary carcinogenesis in transgenic mice model.^15^ Recently, we succeeded in expanding the murine T cell fraction, which contains adiponectin‐expressing Treg, using an experimental thymic model.^16^, ^17^ Notably, these adiponectin‐expressing Treg exhibited cell‐in‐cell phenomenon to thymic stromal nursing cells,^16^ similar to that of previously reported “HOZOT” cytotoxic Treg cells.^18^, ^19^, ^20^ HOZOT cells have been used for tumor‐specific intracellular delivery of “oncolytic adenoviruses” to gastric and colorectal cancer cells through cell‐in‐cell invasion.^21^

In the present study, we examined whether adiponectin‐expressing Treg function as effective adiponectin transporters in TNBC.

## METHODS

### Cells and culture

Two TNBC cell lines, MDA‐MB‐157 and MDA‐MB‐231, and a lobular carcinoma cell line, MDA‐MB‐330, were obtained from the American Type Culture Collection (Manassas, VA, USA). Luminal A‐type breast cancer cell line MCF‐7 was purchased from the Japanese Collection of Research Biosources Cell Bank (Osaka, Japan). The breast cancer cells were passaged for no more than 6 months after resuscitation.

T cells consisting of adiponectin‐expressing Treg were obtained from murine tumors, which mimic human micronodular thymic tumors with lymphoid stroma, and were maintained through coculture with thymic stromal cells, as previously described.^16^, ^17^

Cells were cultured in Dulbecco's modified Eagle's medium‐high glucose (4500 mg/L) (Sigma‐Aldrich) with 10% fetal bovine serum.

In several experiments, cells were labeled using LuminiCell Tracker‐Cell labeling kit (Sigma‐Aldrich).

### Immunofluorescent cytochemistry staining

Immunofluorescence staining was conducted according to a previously described procedure.^22^ Rat anti‐mouse CD4 (clone GK1.5) conjugated with phycoerythrin (PE) (catalog no. 1102040) was purchased from Sony. Rabbit monoclonal antibody against mouse CD25 conjugated with fluorescein isothiocyanate (FITC) (catalog no. 50292‐M08H) was obtained from Sino Biological Inc. Rabbit anti‐adiponectin antibody (cat. no. GTX107737, GeneTex Inc.) and mouse anti‐FOXP3 antibodies (clone 3G3, Proteintech), which react with murine adiponectin and FOXP3, respectively, were also used for staining.

Briefly, the cells were incubated with or without 1:100 diluted antibodies at 4°C for 1 h. The cells were then washed with phosphate‐buffered saline (PBS), and also analyzed using a Guava EasyCyte cell analyzer (Hayward) and a confocal laser scanning microscope (Leica TCS SP8) as previously described.^23^

### Detection of apoptosis

Recombinant human eukaryotic adiponectin was purchased from R&D Systems Inc. The number of cells undergoing apoptosis was quantified using FITC‐conjugated annexin V and propidium iodide (PI: PromoCell GmbH). Briefly, 1 × 10^4^ cells under different culture conditions were harvested, washed, resuspended in binding buffer, mixed with annexin V‐FITC and PI, and analyzed as previously described.^24^

### Immunoblotting

Immunoblotting was performed according to the method described by Towbin et al. with modifications as previously described.^24^, ^25^ Briefly, cell lysates were electrophoresed on sodium dodecyl sulfate‐polyacrylamide gels and electroblotted onto polyvinylidene difluoride membranes (Immobilon‐P Transfer Membrane; Millipore). The membranes were blocked with Block Ace (blocking milk; Yukijirushi) and incubated with rabbit anti‐adiponectin antibody (cat. no. GTX107737, GeneTex Inc.) and anti‐T‐cadherin antibody.^26^ For immunodetection, horseradish peroxidase‐conjugated anti‐rabbit secondary antibody (1:2000; cat. no. 7074); Cell Signaling Technology Inc. and ultrasensitive HRP substrate (TaKaRa Bio) were used. Images were obtained using an Invitrogen iBright 1500 gel imaging system (Thermo Fisher Scientific). After stripping immunoreactivity, the membranes were incubated with an anti‐GAPDH antibody (Sigma) to evaluate the input proteins.

### Coculture of TNBC cells and adiponectin‐expressing Treg

CD4‐ and CD25‐positive T cells were harvested using an SH800S cell sorter (Sony Biotechnology). Subsequently, adiponectin‐expressing Treg were cocultured with MDA‐MB‐157 or MDA‐MB‐231 cells. Time‐lapse images of the cells were acquired using a CytoWatcher (ATTO) or confocal laser scanning microscope (Leica TCS SP8).

## RESULTS

### Harvest of adiponectin‐expressing Treg

**FIGURE 1:**
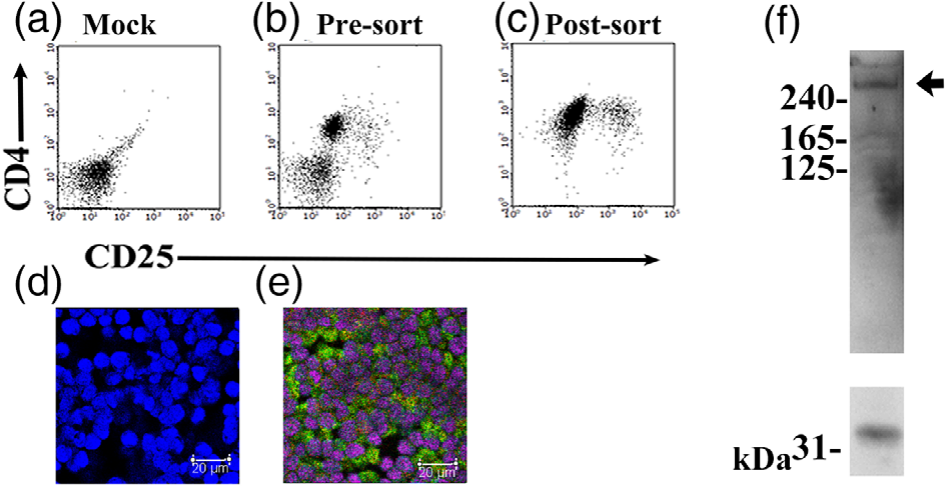
Representative results of adiponectin‐expressing Treg harvesting. Lymphocytes were obtained from a culture of cells from a previously developed murine model of a human micronodular thymic tumor with lymphoid stroma.^16^, ^17^ Control nonstained cells are shown in (a). Lymphoid cells were partially stained on the cell surface with anti‐CD4 and anti‐CD25 antibodies (b). CD4‐ and CD25‐positive lymphoid cells were sorted using an SH800S cell sorter (c). Subsequently, the cells were immunocytostained with antibodies. Nonimmunostained cells (stained with 4′,6‐diamidino‐2‐phenylindole [DAPI]) are shown in (d). Sorted cells exhibited FOXP3 and adiponectin immunoreactivity. In (e), the green signal indicates adiponectin immunoreactivity and the pink color indicates the merging of red FOXP3 immunoreactivity and blue DAPI staining. Scale bar, 20 μm. The immunoblotting results indicated that the sorted cells expressed high molecular weight (HMW) adiponectin, as indicated by the arrow (f; upper column). The glyceraldehyde‐3‐phosphate dehydrogenase (G3PDH) band is shown (f; lower column).

The detailed characterization of the murine tumor model from which we obtained adiponectin‐expressing Treg cells has been previously documented.^16^, ^17^ Briefly, the murine model exhibited thymic tumors composed of thymic epithelioid tumor cells with abundant lymphoid stroma. As shown in Figure S1, the lymphoid stroma partially contained adiponectin‐expressing and FOXP3‐positive Treg cells. Using CD4‐ and CD25‐positive sorting, we successfully harvested adiponectin‐expressing Treg cells that exhibited FOXP3 nuclear staining. Immunoblotting also demonstrated that the sorted Treg cells expressed HMW adiponectin. The representative results are shown in Figure 1.

### Limited effect of soluble adiponectin on MDA‐MB‐157 cells

**FIGURE 2:**
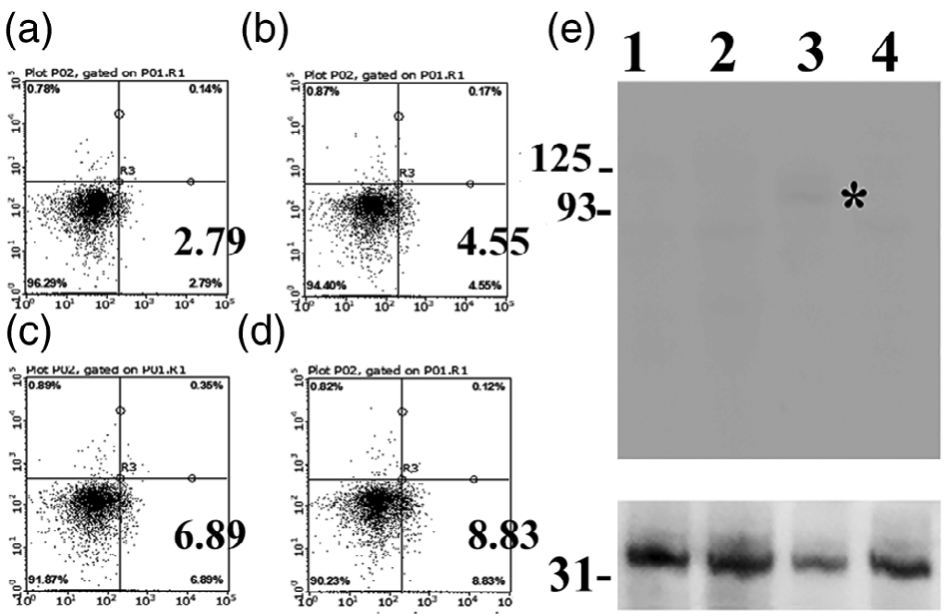
Limited effect of soluble adiponectin on apoptosis induction on triple negative MDA‐MB‐157 breast cancer cells. (a)–(d) MDA‐MB‐157 cells were incubated with eukaryotic recombinant adiponectin at various concentrations (a:0, b:0.05, c:0.5, d:5 μg/mL) for 36 h. Thereafter, the cells were examined using an annexin V‐PI assay. Even under 5 μg/mL adiponectin, less than 10% of cells were apoptotic. (e) *Indicates a 105 kDa T‐cadherin protein band, which is characterized as a mature receptor of high molecular weight (HMW) adiponectin. A weak 105 kDa T‐cadherin protein band was seen in lane 3 (MDA‐MB‐157), but not in lanes 1 (MDA‐MB‐330), 2 (MDA‐MB‐231), or 4 (MCF‐7). The glyceraldehyde‐3‐phosphate dehydrogenase (GAPDH) band is shown in the lower column.

The representative results of soluble adiponectin induced apoptotic effect on MDA‐MB‐157 cells are shown in Figure 2. MDA‐MB‐157 cells underwent apoptosis in a dose‐dependent manner after treatment with soluble adiponectin. However, a minor proportion of MDA‐MB‐157 cells, that is, less than 10% of cells underwent apoptosis. Immunoblotting revealed a weak T‐cadherin band in MDA‐MB‐157 cells.

### Cell‐in‐cell phenomenon of adiponectin‐expressing Treg in TNBC cells

**FIGURE 3:**
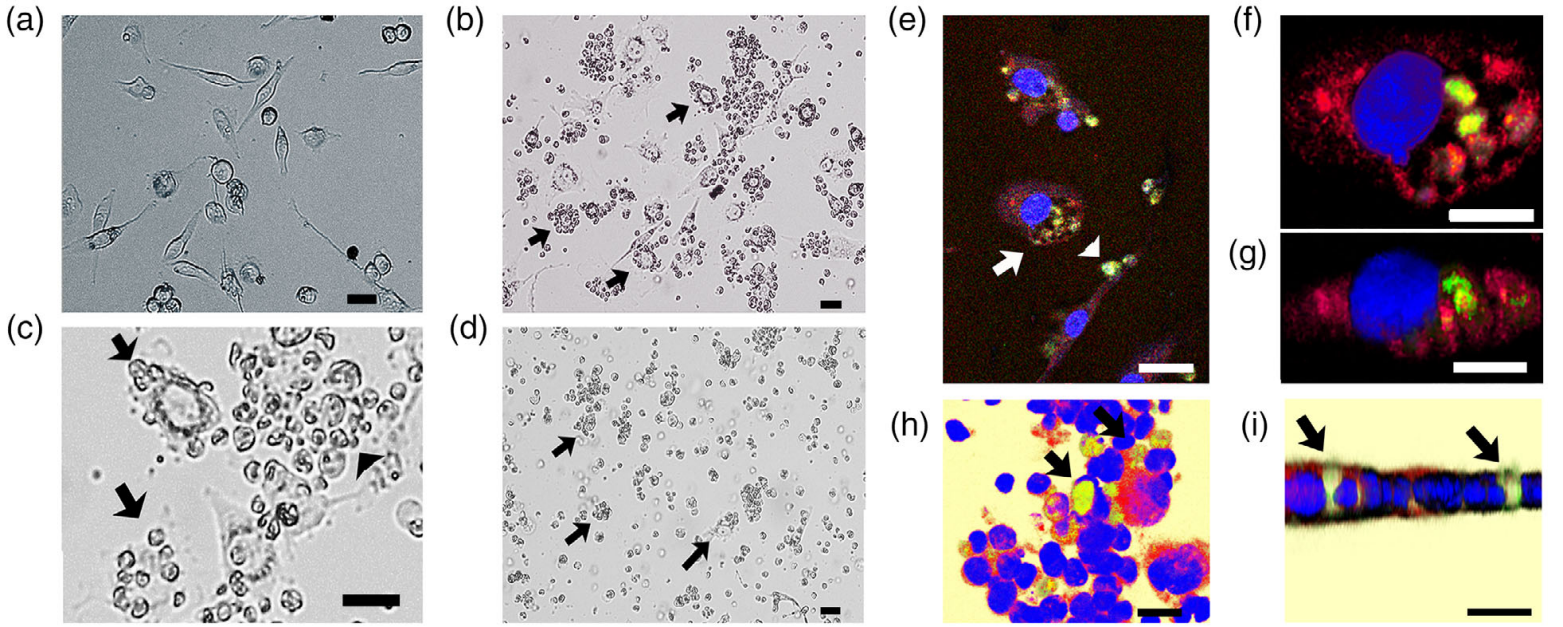
Triple‐negative breast cancer cells exhibited cell death after engulfing adiponectin‐expressing Treg. MDA‐MB‐157 cells (a) were occulted due to adiponectin‐expressing Treg. After 16 h, numerous adiponectin‐expressing Treg attached to MDA‐MB‐157 cells (b). Note the integration of adiponectin‐expressing Treg into the cytoplasm (b and c, arrow) and destruction (c, arrowhead) of MDA‐MB‐157 cells. After 64 h of coculture, MDA‐MB‐157 cells exhibited cell death (d, arrow; insert indicates cell debris). Confocal laser imaging confirmed cytoplasmic integration, that is, the cell‐in‐cell phenomenon (e, f, and g) in MDA‐MB‐157 cells. MDA‐MB‐157 cells, the cytoplasm of which was stained red, engulfed adiponectin‐expressing Treg stained green. Note the attachment (e, arrowhead) and cytoplasmic integration (e, arrow) of adiponectin‐expressing Treg into MDA‐MB‐157 cells. Horizontal and vertical cell‐in‐cell images are shown in (f) and (g), respectively. MDA‐MB‐231 cells also demonstrated the cell‐in‐cell phenomenon by engulfing adiponectin‐expressing Treg as indicated by arrows in the horizontal (h) and vertical (i) images. The scale bar indicates 50 μm.

Adiponectin‐expressing Treg were cocultured with MDA‐MB‐157 and MDA‐MB‐231 cells. Notably, we observed a cell‐in‐cell phenomenon or the engulfment of adiponectin‐expressing Treg in many MDA‐MB‐157 cells. MDA‐MB‐231 cells also exhibit cell‐in‐cell characteristics to a less extent. Notably, almost all the MDA‐MB‐157 cells were destroyed after engraftment with adiponectin‐expressing Treg. The representative results are shown in Figure 3.

## DISCUSSION

Here, we demonstrated that using CD4 and CD25 sorting, adiponectin‐expressing Treg cells can be successfully harvested (Figure 1a–e) from cultured cells of an experimental murine thymic tumor model (Figure S1). Notably, these Treg cells expressed HMW adiponectin (Figure 1f). Recombinant adiponectin failed to induce sufficient apoptosis in MDA‐MB‐157 triple negative breast cancer cells (Figure 2a–d), possibly due to the downregulation of T‐cadherin, a receptor of HMW adiponectin, as previously reported.^4^, ^6^, ^7^ Interestingly, adiponectin‐expressing Treg cells exhibited cell‐in‐cell phenomena directed towards MDA‐MB‐157 breast cancer cells, which resulted in the death of the MDA‐MB‐157 cells (Figure 3).

The cell‐in‐cell phenomenon was observed in tumor cells as early as 1891 by Steinhaus;^27^ thereafter, it has been documented in various cancers, including TNBC (see Review in Fais and Overholtzer^28^; Zhang et al.^29^).

Previously, we noted that adiponectin‐expressing Treg exhibit the cell‐in‐cell phenomenon to thymic stromal nursing cells.^16^ In this study, we demonstrated that adiponectin‐expressing Treg attached to and integrated into TNBC cells. As shown in Figure 3, most TNBC cells died after the engulfment of adiponectin‐expressing Treg.

Previous studies have reported that a Treg cell line, designated HOZOT, exhibits the cell‐in‐cell phenomenon in several cancer cells. HOZOT cells were established from human umbilical cord blood through coculture with mouse stromal cells and were characterized as a cytotoxic Treg line with cell‐in‐cell activity.^18^, ^19^, ^20^ HOZOT cells demonstrated tumor‐specific intracellular delivery of “oncolytic adenoviruses” to gastric and colorectal cancer cells through cell‐in‐cell invasion.^21^

Adiponectin is believed to play a protective role against carcinogenesis in various types of cancers.^4^ Therefore, adiponectin‐based therapy has been proposed as a potential pharmacological approach in both cancer prevention and management. Nevertheless, the intrinsic limitations of adiponectin, that is, the HMW of its active form and reduced stability, have hampered its therapeutic application in clinical settings, including its use for the treatment of cancer. Moreover, T‐cadherin, a receptor of HMW adiponectin, is downregulated in many cancers, including breast cancer.^6^, ^7^ Notably, the Treg cells in the present study were shown to have a high level of HMW adiponectin in the cytoplasm (Figure 1). Therefore, engulfment of adiponectin‐expressing Treg cells may enable the enforced uptake of adiponectin to malignant tumors, whether or not adiponectin receptors are present.

Adiponectin‐expressing Treg cells were obtained from T cells, which were maintained by coculture with murine thymic stroma cells.^16^ Without direct cellular contact with thymic stromal cells, T cells undergo apoptosis.^17^ Some Treg cells are also known to be engulfed by hepatocellular cell carcinoma cells.^30^ We speculate that the adiponectin‐expressing Treg in the present study may be a relative of HOZOT or other Treg subtypes that have cell‐in‐cell activity. However, further studies are necessary to verify this hypothesis and to unravel the molecular mechanisms that participate in the Treg‐mediated cell‐in‐cell phenomenon.

In conclusion, in this study, we established that novel adiponectin‐expressing Treg cells exhibit a cell‐in‐cell phenomenon, subsequently causing cell death in TNBC cells. We believe that they function as effective adiponectin transporters in TNBC cells.

## AUTHOR CONTRIBUTIONS

CS and TT designed and conceived the study. YC, TH, HH, and YH performed the experiments. YK, MF, and NM interpreted the data. All authors participated in the data analysis. All authors read and approved the final manuscript.

## CONFLICT OF INTEREST STATEMENT

The authors declare no competing interests.

## Supporting information


**FIGURE S1.** The experimental murine thymic tumor was composed of thymic epithelial nest (H&E‐stained tissues; white arrow) and lymphoid stroma (black arrow). Notably, lymphocytes of the lymphoid stroma partially exhibited nuclear FOXP3 and cytoplasmic adiponectin immunoreactivities (indicated by white arrow). Scale bars, 50 μm.
