# Supplementary figures and images for: Potential activity of adiponectin‐expressing regulatory T cells against triple‐negative breast cancer cells through the cell‐in‐cell phenomenon
Source: Thorac Cancer. 2023 May 23;14(20):1941–5. doi: 10.1111/1759-7714.14940 (PMC10344742; doi:10.1111/1759-7714.14940)

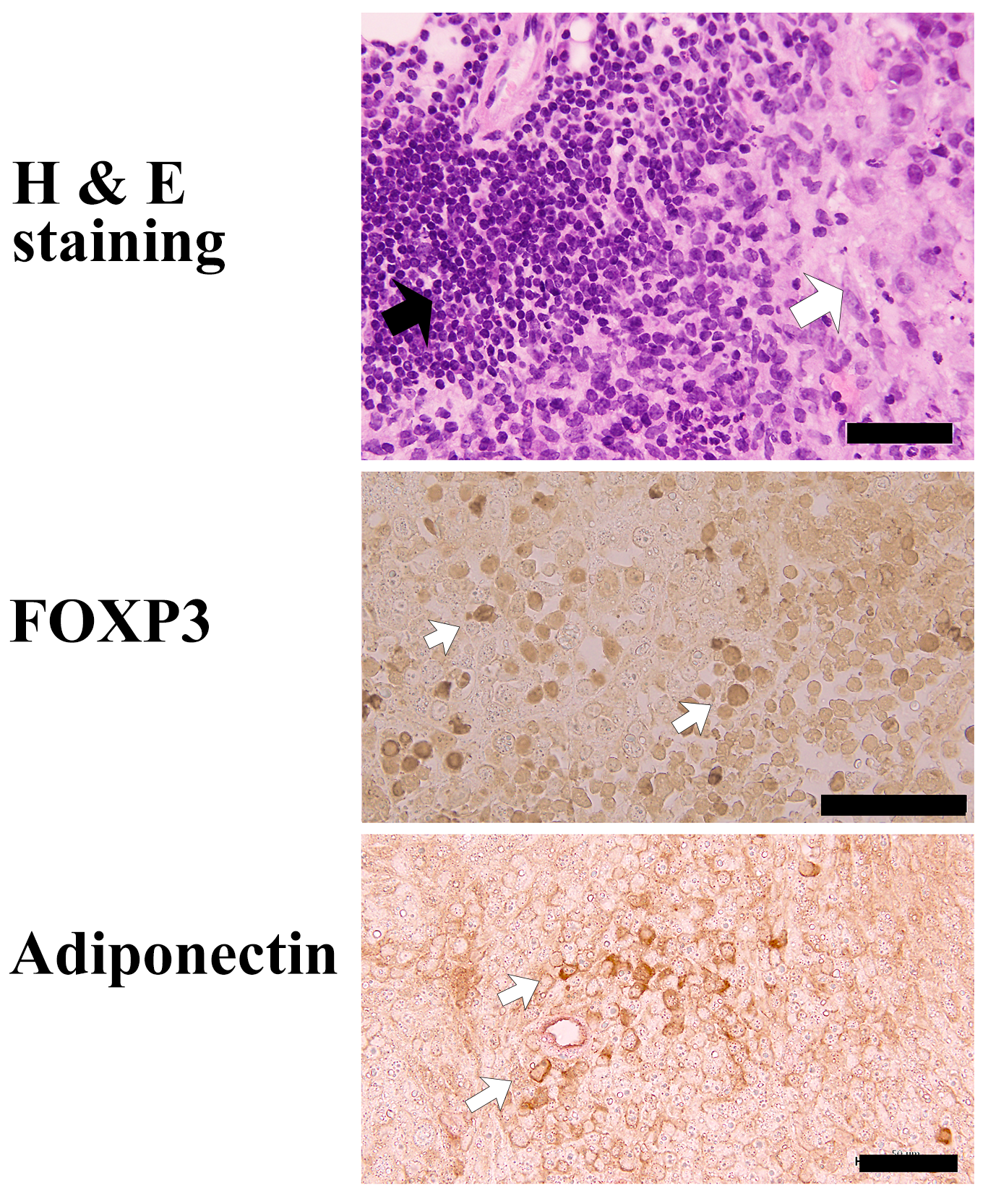

Supplement: Supplementary file 1 — FIGURE S1. The experimental murine thymic tumor was composed of thymic epithelial nest (H&E‐stained tissues; white arrow) and lymphoid stroma (black arrow). Notably, lymphocytes of the lymphoid stroma partially exhibited nuclear FOXP3 and cytoplasmic adiponectin immunoreactivities (indicated by white arrow). Scale bars, 50 μm. [file TCA-14-1941-s001.tif]
